# Implementation of community health care services to counter the SARS-CoV2 pandemic

**DOI:** 10.1186/s12913-024-10607-x

**Published:** 2024-02-01

**Authors:** Assunta De Luca, Luca Provvidenti, Mario Muselli, Giacinto Di Gianfilippo, Massimiliano Angelucci, Michele Ciro Totaro, Mauro Pitorri, Marzia Marcelli, Marinella D’Innocenzo, Maria Scatigna, Riccardo Mastrantonio, Stefano Necozione, Leila Fabiani

**Affiliations:** 1 Local Health Unit Rieti, Rieti, Italy; 2 Department of Life, Health and Environmental Sciences, University of L’Aquila, L’Aquila, Italy

**Keywords:** Public health, SARS–CoV-2, Hospitalization, Emergency department, Local health policy, Community health care services

## Abstract

**Background:**

The COVID-19 pandemic has ravaged many countries worldwide since December 2019. The high infection rates, and the need for health care assistance for individuals with comorbidities, strained the national health care systems around the world. Outbreak peaks increased the burden on hospitals that where perceived as high-risk places by people, who often decided to cancel or defer hospital visits. Thus, Italian Local Health Authorities had to develop new organizational models to meet the increased health care needs of the population. The aim of this study is to assess the impact of strengthened community health services on the hospital burden.

**Methods:**

We analysed the number of Emergency Department access at the Hospital De Lellis covered by the Local Health Authority in Rieti, from March 2020 to November 2021. We then assessed the effects of community health services: the Special District Continuing Care Units (SDCUs) and the the COVID hub, on the COVID-19-related ED access, admission and mortality rates. A Chi-squared test for trend and three multivariable logistic regression models were used to investigate the trends and the possible predictors of COVID ED access, COVID hospital admissions, and deaths.

**Results:**

Being male (OR = 1.41, CI95% 1.05–1.90; *p* = 0.022) and older age (OR = 1.03, CI95% 1.02–1.04; *p* < 0.0001) increase the likelihood of hospitalisation for Sars-CoV-2. The implementation of the nursing and medical SDCUs contributed to reducing COVID-19-related deaths (OR = 0.09, CI95% 0.03–0.29; *p* < 0.0001). The simultaneous implementation of the COVID hub and of the nursing SDCUs had a synergistic effect in reducing the likelihood of hospitalisation (OR = 0.24, CI95% 0.09–0.65; *p* = 0.005). The subsequent implementation of the medical SDCUS has further contributed to lowering the admission rates. These protective effects persisted also after potential cofounders, such as age, sex, clinical condition on admission, and the immunisation status, were adjusted.

**Conclusions:**

These measures have helped in the management of patients in a complex context such as that of a pandemic by reducing the hospital load and playing an important role in the management of the pandemic. Further studies could assess the transferability of this model in a non-pandemic context.

## Introduction

The COVID-19 pandemic has ravaged many countries worldwide since December 2019. The high infection rates, the high transmissibility, the need for health care assistance for individuals with comorbidities, have strained the national health care systems around the world in unprecedented ways. In order to alleviate the hospital burden and to meet the health care needs of the population, new organizational models were implemented worldwide for an optimal resource allocation [1].

During the first pandemic phase, when COVID-19 vaccines were not yet available, the population had a high risk perception [2]. Moreover, many people avoided medical care for fear of contracting COVID-19 infection in the hospital. Several studies have shown that Emergency Department (ED) access due to diseases other than COVID-19 drastically decreased [3, 4]. Such evidence might have an impact on the delivery and management of public healthcare services [5].

Some authors agree that this evidence might have positive effects: for example, the study conducted by Bhatt et al. [6] highlights a decline in the hospitalization rates and in the hospital length of stay for non–COVID-19 illnesses, with no significant variation in the in-hospital mortality rates. The study by Pinnarelli et al. [7] also suggests that the overall reduction in ED access might have positive consequences, such as an increased appropriateness of hospital admissions-.

However, other studies such as those by Muselli et al. [8] and by Arniani et al. [9], agree that the effects of the reduction in hospital admissions on population health are difficult to be assessed, both in terms of a possible increase in the out-of-hospital mortality rates, and in terms of a possible undiagnosed silent damage that might have long-term impacts on the health of the individual.

Therefore, the Healthcare System should provide better coverage and access to medical services, for example by increasing the effectiveness of the community health services. This appears to be a necessary action when the burden on hospitals is severe and they are perceived as risk places, thus resulting in deferred or cancelled visits.

During the SARS-CoV-2 pandemic, the implementation of the community-based outpatient services may be one possible scenario to avoid the collapse of the Healthcare System and to ensure that COVID-19 patients receive the appropriate medical assistance. In their study, Moghadasa et al. [10] projected the number of ICU beds required at the peak of the outbreak in the USA in case contact-tracing had failed. When R_0_ = 2.5, treatment of critically ill individuals at the outbreak peak would require 3.8 times more ICU beds than exist in the United States. When R_0_ = 2, twice as many ICU beds would be required at the peak of outbreak. In addition, the authors estimated that self-isolation by 20% of cases within 24 h after symptom onset would reduce the peak number of ICU beds needed by 48.4% and 73.5%, respectively.

The study by Patel et al. [11] has also highlighted some critical aspects in the management of the pandemic. For example, hospital overcrowding has led to a shortage of beds, partly caused by the difficulty of finding a suitable setting for discharge. This observation suggests the need for planning emergency preparedness to alleviate the pressure on hospitals during future health crises. The authors highlight the lack of community care facilities in the USA that would admit patients regardless of their test results, thus avoiding hospital overcrowding.

This study aims to assess whether an organizational approach designed to alleviate the hospital burden by strengthening the delivery of community health services and of home health care services contributed to managing COVID-19 outbreak peaks, thus ensuring more accurate diagnosis and reducing hospitalisation and mortality.

## Materials & methods

This is a retrospective study of the population who referred to the Local Health Authority in Rieti, Italy, from March 2020 to November 2021. The Local Health Authority in Rieti reported an average assisted population of 151,000 subjects during the study time with an equal distribution by gender. The average age of assisted was 47.3 years and the median value 50 years [12].

To assess the impact of the community health services implemented to reduce the spreading of the virus, data were collected from several community-based health care facilities. The data related to the ED access to the Hospital San Camillo de Lellis (Local Health Authority in Rieti) were extracted from the Emergency Department Information Systems (EDIS). Hospital admission records of the same hospital were extracted from the Hospital Information System (HIS). Then, the data were matched through deterministic record linkage analysis using the tax and social security number of the patient.

Hospital access from 1 March 2020 to 24 November 2021 were taken into account. Data included: age, sex, access date, ED triage, evaluation of the severity (admissions, transfers, home discharges) and SARS-CoV-2 test result. Hospital access have been distinguished between related to COVID-19 and not related to COVID-19 based on the positivity of the SARS-CoV-2 test. Triage colour tags were dichotomized into nondeferrable emergency (yellow and red) and deferrable emergency as an indicator of emergency severity. We analysed the immunisation registry to verify the COVID-19 immunisation status of each patient accessing the ED. The data refer to the ED access and not to the patients; therefore, if the same subject was re-admitted during the study period, they were taken into account more than once.

Data on COVID-19 patients who had to be hospitalised included patient outcome data (home discharge, transfer, death).

**Fig. 1:**
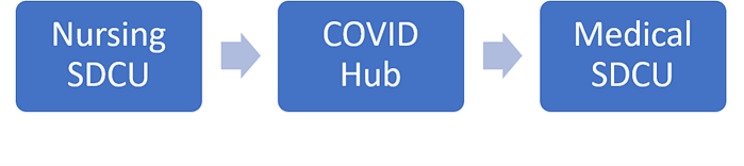
Timeline of the measures implemented

Community health services included the implementation of Special District Continuing Care Units (SDCUs) and the implementation of a COVID hub specialized in the local management of COVID-19 cases. These measures were implemented at later stages (Fig. 1). The SDCUs are special mobile teams that, on the recommendation of the family doctor or of the paediatrician, assess confirmed or suspected COVID-19, who were discharged from the ED or from the hospital to home or to community health care facilities. The implementation of the SDCUs included two stages: first, only community nursing services were delivered, then nurses were accompanied by physicians. In order to assess the community health care services, we used an ordinal variable accounting for the various levels of the measures implemented, from the least to the most complex level: no measure implemented (0); nursing SDCU (1); nursing SDCU and COVID hub (2); nursing and medical SDCUs and COVID hub (3 -highest level of measure implementation). Since the SDCUs and the COVID hub were implemented simultaneously, these two measures were grouped into one variable (Fig. 1).

A Chi-squared test for trend was performed with respect to the ordinal variable of the community health services to analyse the proportion of COVID-19-related access amongst the overall ED access, the proportion of COVID-19-related access resulting in admission, and the proportion of the COVID-19 hospitalised patients. COVID ED access, COVID hospital admissions, and COVID related mortality considered dependent variables for separate multivariable logistic regression models. In each multivariable model, the independent variables included age, gender, emergency severity (deferrable emergency/nondefferable emergency), occurrence of positives, community health services implemented (none/nursing SDCU/nursing SDCU and COVID hub/nursing and medical SDCUs and COVID hub), and immunization status. Occurrence of positives refers to the number of COVID-19 cases in the province. It was used to keep track of the virus trend and it was classified into quartiles as follows: <27; 27–159; 160–607;>607. Significance level was set to 0.05.

## Results

During the study period, a total of 38,873 hospital visits were recorded, of which 1,228 (3.16%) were patients with lab-confirmed COVID-19 or with COVID-19 related symptoms. Table 1 reports the ED access due to COVID-19 from 2020 to 2021, by age, sex, emergency severity, and proprortion of hospital admissions. In 2020, a total of 18,804 admissions to the ED were recorded, of which 629 (3.35%) were COVID-19 related admissions; in 2021, 20.069 admissions to the ED were recorded, of which 599 (2.98%) were COVID-19 related admissions.

**Table 1:** COVID-19 Emergency Department visits from 2020–2021 by admission, age, sex, and triage status

	2020	2021
**No. COVID admissions**	629	599
**Hospitalisations, no (%)**	470 (74.72%)	421 (70.28%)
**Age, mean ± SD**	70.98 ± 18.02	65.17 ± 17.99
**Sex, n (%)**		
- Males	349 (55.48%)	345 (57.60%)
- Females	280 (44.52%)	254 (42.40%)
**Nondeferrable emergencies, n (%)**	408 (65.28%)	352 (59.26%)

Table 2 highlights the differences in the COVID-19 admission rates in 2020 and in 2021, by age, sex, and death. In 2020, 74.72% of the COVID-19 ED visits resulted in hospitalizations, whereas in 2021 this proportion dropped to 70.28%.

**Table 2:** COVID-19 hospital admissions in 2020 and in 2021 by age, sex, and death

	2020	2021
**No. COVID hospitalisations**	470	421
**Age, mean ± DS**	70.03 ± 15.09	68.73 ± 14.01
**Sex**		
- Males	270 (57.45%)	246 (58.43%)
- Females	200 (42.55%)	254 (41.57%)
**Death rates**	136 (28.94%)	89 (21.14%)

By taking into account the community health services that were implemented before the COVID-19 vaccination campaign was launched, we assessed the changes in the outcomes considered (Table 3). The implementation of the nursing SDCUs led to a reduction in the ED access due to COVID-19. However, during the period coinciding with the implementation of the COVID-19 contact tracing hub, we registered an increase in the access proportion, which then declined (*p* < 0.0001). Overall, the health care services resulted in a reduction in COVID-19 death proportions, even if not statistically significant.

**Table 3:** COVID-19 ED access amongst the total ED access, COVID-19 hospital admissions amongst the total COVID-19 ED access, and COVID-19 death rates amongst the hospital admissions in relation to the community health services

Community health care service	Total ED access	COVID-19 ED access	COVID-19 hospital admissions	Death rates
**None**	1,485	52 (3.5%)	41 (78.85%)	15 (36.59%)
**Nursing SDCU**	12,888	104 (0.81%)	79 (75.96%)	8 (10.13%)
**Nursing SDCU + COVID hub**	4,431	473 (10.67%)	350 (74.00%)	113 (32.29%)
**Nursing SDCU + COVID hub**	4,744	377 (7.95%)	304 (80.64%)	69 (22.70%)
***p***-value*	-	< 0.0001	0.2304	0.4947

^*^ Chi square test for trend

In order to assess the impact of the community health services on the number of ED access due to COVID-19, a multivariable logistic regression analysis was performed after excluding potential confounders (Table 4). Confounders included age, sex, immunisation status, number of COVID-19 cases registered on the day of the hospital admission. Increasing age, male sex, urgent triage category, and an increased rate of positive cases were higher predictors of COVID-19 related visits to hospital. On the other hand, the implementation of the SDCU, of the COVID hub, and being vaccinated, represented protective measures.

**Table 4:** Odds ratio of the associations between the community health services and COVID-19 ED access

	Odds Ratio	95% C.I.	*p*-value
**Age**	1.02	1.01–1.02	< 0.0001
**Sex**			
- **Males**	Ref.		
- **Females**	0.87	0.76–0.99	0.031
**Emergency severity**			
- **Deferrable emergencies**	Ref.		
- **Nondeferrable emergencies**	2.32	2.02–2.68	< 0.0001
**Occurrence of positives**			
- **I quartile (< 27)**	Ref.		
- **II quartile (27–159)**	3.72	2.41–5.73	< 0.0001
- **III quartile (160–607)**	9.12	5.97–13.94	< 0.0001
- **IV quartile (> 607)**	49.13	31.45–76.75	< 0.0001
**Community health care services**			
- **None**	Ref.		
- **Nursing SDCU**	0.16	0.11–0.23	< 0.0001
- **Nursing SDCU and COVID hub**	0.20	0.13–0.29	< 0.0001
- **Nursing and medical SDCUs and COVID hub**	0.10	0.07–0.14	< 0.0001
**Vaccinated**	0.82	0.72–0.94	0.005

To assess the impact of the community health services on the number of hospital admissions, a multivariable logistic regression analysis was performed accounting for the same variables as in the above model (Table 5). Increasing age, male sex, urgent triage category, were found to be higher predictors of hospital admission. On the other hand, hospitalisation rates seemed to decline only after that the COVID hub and the medical SDCUs had been implemented.

**Table 5:** Odds ratio of the associations between the community health services and likelihood of COVID-19 hospital admission

	Odds Ratio	95% C.I.	*p*-value
**Age**	1.03	1.02–1.04	< 0.0001
**Sex**			
- **Males**	Ref.		
- **Females**	0.71	0.53–0.95	0.022
**Emergency severity**			
- **Deferrable emergencies**	Ref.		
- **Nondeferrable emergencies**	2.10	1.54–2.86	< 0.0001
**Occurrence of positives**			
- **I quartile (< 27)**	Ref.		
- **II quartile (27–159)**	0.86	0.30–2.48	0.778
- **III quartile (160–607)**	1.01	0.36–2.86	0.985
- **IV quartile (> 607)**	2.70	0.92–7.95	0.072
**Community health care services**			
- **None**	Ref.		
- **Nursing SDCU**	0.56	0.22–1.47	0.245
- **Nursing SDCU and COVID hub**	0.24	0.09–0.65	0.005
- **Nursing and medical SDCUs and COVID hub**	0.26	0.10–0.68	0.007
**Vaccinated**	1.08	0.79–1.49	0.624

Finally, to assess the impact of the community health services on the death rates, a multivariable logistic regression analysis was conducted with same variables as in the previous models after excluding potential confounders (Table 6). Increasing age, urgent triage category, and COVID-19 self-isolation cases > 159 were higher predictors of death. The community health services and vaccination significantly increased protection against death.

**Table 6:** Odds ratio of the associations between community health services and likelihood of COVID-19 related mortality following ED access

	Odds Ratio	95% C.I.	*p*-value
**Age**	1.07	1.06–1.09	< 0.0001
**Sex**			
- **Males**	Ref		
- **Females**	0.82	0.58–1.14	0.235
**Emergency severity**			
- **Deferrable**	Ref.		
- **Nondeferrable**	2.12	1.41–3.18	< 0.0001
**Occurrence of positives**			
- **I quartile**	Ref.		
- **II quartile**	2.35	0.40–13.92	0.344
- **III quartile**	9.69	1.53–61.40	0.016
- **IV quartile**	36.66	5.01–263.02	< 0.0001
**Community health care services**			
- **None**	Ref.		
- **Nursing SDCU**	0.09	0.03–0.29	< 0.0001
- **Nursing SDCU and COVID hub**	0.06	0.02–0.22	< 0.0001
- **Nursing and medical SDCUs**	0.07	0.02–0.25	< 0.0001
**Vaccinated**	0.42	0.29–0.61	< 0.0001

## Discussion and conclusions

The world was unprepared for the COVID-19 pandemic. In Italy, the novel Sars-CoV-2 caused a public health crisis. Several urgent measures have been implemented since the World Health Organization declared the Coronavirus disease outbreak a public health emergency of international concern [13]. The pandemic exhibited distinct temporal phases, each presenting unique challenges. The implementation of community health services coincided with these phases, complicating the attribution of outcomes solely to the intervention. The effectiveness of our measures should be considered in light of the dynamic nature of the pandemic. In the early stage of the emergency, response to contain and manage COVID-19 was provided by hospitals only whereas the importance of the community health care services was initially underestimated. Thus, in addition to managing ICU bed occupancy, appropriate measures for the organization of the community health services had to be implemented. In order to meet the increased health care needs, the Italian health authorities had to adapt and/or reorganize the health care personnel. It must also be considered that external factors, such as changes in public behavior, governmental policies, and the concurrent rollout of vaccination campaigns, likely interacted with our intervention. These external elements may have influenced healthcare-seeking behavior and the overall trajectory of COVID-19 outcomes in the community.

We observed an improvement between the two years studied. Emergency department access due to COVID declined from 629 to 599, the proportion of COVID-19 hospitalisations decreased from 74.72% in 2020 to 70.28% in 2021. In addition, we observed a decreased disease severity among COVID-19 patients who attended the ED as nondeferrable emergencies declined from 65.28 to 59.26%, and a decreased mortality rate (89 in 2021 vs. 136 in 2020).

On the basis of the results obtained, it appears that the implementation of the nursing SDCU helped to observe a reduction in the number of COVID-19-related visits, admissions, and deaths. Death rate declined from 30.77 to 9.62%. Following the implementation of the COVID contact tracing hub, we observed a higher number of COVID-19-related ED access, accounting for 10.67% of the overall ED access. This percentage might be explained as an increased selection of the patients accessing the hospital. Indeed, along with the launch of the vaccination campaign and the decreased mortality rate, contact tracing and counselling carried out by the COVID-19 hub might have contributed to ensuring an increased appropriateness of ED access. As physicians were allocated to the medical SDCU, we observed a further decline in the proportion of COVID-19 ED access (7.95% amongst the total access), and in the mortality rate (22.70%).

The initiation of the COVID-19 vaccination campaign represents a significant milestone. Following the vaccination campaign, we observed a significant decline in the outcomes considered: -77.9% in the COVID-19 ED access, -84,9% in the COVID-19 hospital admissions, and − 90.9% in the death rates.

However, these measures were implemented in different phases of the pandemic that was characterized by a different trend of the infection curve and different models of clinical management. Therefore, we deemed appropriate to build regression models accounting for this confounder including occurrence of positives, implemented community health care services as covariates, and vaccination. The results of the first model suggest that the measures implemented were effective to reduce COVID-19 ED access. In addition, the various community health services implemented to prevent the spread of the pandemic seem to have a long-term and increasing effect against hospitalisation. In fact, although the decreased likelihood of hospitalisation through the implementation of the nursing SDCU is not statistically significant, the implementation of the COVID hub and of the SDCUs has reduced the likelihood of hospitalisation in a statistically significant way (OR = 0.24), as confirmed by the implementation of the medical SDCUs (OR = 0.26).

In agreement with the research conducted by Garg et al. [14], being males and an older age increase the likelihood of hospital access, of admission, and of mortality due to COVID-19 in a statistically significant way. About the mortality rate, the community health services resulted to be immediately effective (OR = 0.09) and showed a long-term and increasing effect, confirmed by the subsequent implementation of new health services.

Our study extends the projections made by Moghadasa et al., who focused on the number of ICU beds required during the peak of the outbreak. We build on this by examining the impact of community health services on reducing ED access and hospital admissions. Similarly, the observations made by Patel et al. regarding hospital overcrowding and the need for community care facilities align with our emphasis on the importance of community health services in alleviating the pressure on hospitals.

The main limitation of our study is the lack of contextualization of the data reported within the different community health services implemented, the quarantine measures and the restriction rules that, as highlighted by the research conducted by Guaitoli et al. [15], might play an important role in reducing the infection risk across the different geographical areas and in the different historical moments. Another limitation is the lack of detailed comorbidity data that may impact the comprehensiveness of our findings, as comorbid conditions can play a significant role in shaping the severity of COVID-19 outcomes. Moreover, despite our efforts to control for confounding variables in our regression models, it is important to note that unmeasured confounders may exist. These variables, not accounted for in our analysis, could potentially influence the observed associations between community health services and COVID-19 outcomes. Finally, while our study provides insights into the effectiveness of community health services in Rieti, Italy, caution is warranted when generalizing these findings to other settings or populations. The unique characteristics of the study region and the specific implementation of health services may limit the applicability of our results beyond the studied context.

In conclusion, our findings suggest that the community health services contributed to reducing COVID-19 related hospital admissions and thus the burden on the hospital. Although the vaccination campaign has played a fundamental role, the implementation of other effective measures has also contributed to protecting public health and to reducing the mortality rate.

The relocation of the delivery of community health care services has represented a key factor during the pandemic, which might be applied to more sectors of the Public Health, not only during emergencies.

## Data Availability

The datasets generated and/or analysed during the current study are not publicly available due to privacy or ethical restrictions.

## Abbreviations

SDCU: Special District Continuing Care Units
ED: Emergency Department
EDIS: Emergency Department Information Systems
HIS: Hospital Information System
